# The Clinical Society of Louisville

**Published:** 1891-04

**Authors:** 


### THE CLINICAL SOCIETY OF LOUISVILLE.

Stated meeting, January 13, 1891.

THOS. P. SATTERWHITE, M. D., President, in the chair.

DR. L. S. MCMURTRY presented a specimen from a case of extra-uterine pregnancy, with the following history:

MRS. S. E. M., aged twenty-seven years, married nine years. Eight years ago she suffered an abortion at three months, has had uterine disease ever since, and has been sterile. She missed the menstrual period in November, and on December 7th called to see her physician, Dr. George W. Griffiths. Her complaints were of general abdominal pain and discomfort. She again called on Dr. Griffiths on December the 11th. On the 13th, two days later, she had a violent paroxysm of pelvic pain localized on the right side. Dr. Griffiths saw her soon afterward and administered a dose of morphia. She was relieved for the time. On the evening of the 18th Dr. Griffiths summoned me to meet him in consultation, and expressed the belief that abdominal section was indicated. The abdomen was swollen and tender with increasing peritonitis. There was a bloody flow from the uterus. The patient was pallid as from *post-partum* hemorrhage. Vaginal examination showed the uterus pushed to the left side and the pelvis choked with effusion. The pulse was 134, small, the pulse of hemorrhage. The bowels had not acted in four days. We gave an energetic purgative, and arranged for operation the following morning.

Early on the morning of the 19th I opened the abdomen. Dr. J. W. Guest gave ether and Dr. Griffiths assisting. On opening the peritoneum a large quantity of blood flowed out over the table. More than a gallon of blood-clot was removed. The fetal ball was on the right side. The right appendage was tied off close to the uterus, the cavity irrigated with warm distilled water, a glass drainage tube placed, and the abdomen closed. When put on the table the pulse was 140 and quite feeble. The appendage on the opposite side was not removed, as I feared to prolong the operation. The operation was concluded in thirty minutes.

The specimen is of great interest. You will recognize here the ovary, and here the ruptured fallopian tube and the fetal envelopes. From this poured the fearful hemorrhage, which invariably ends in death if not arrested by surgical interference.

This is the first case of extra-uterine pregnancy, so far as I can learn, operated upon in Louisville by abdominal section at the time of rupture. The success of the case is due to Dr. Griffiths' recognition of the gravity of the situation, and advice for immediate operation.

Ectopic gestation is a very common accident. Hundreds of women perish annually from this cause because it is not recognized. Dr. Formad, the well-known pathologist of the University of Pennsylvania, as coroner's physician for Philadelphia, states that in one year he found *post mortem* nineteen cases of ruptured ectopic pregnancy unrecognized. The symptoms are those of shock, internal hemorrhage and peritonitis. The patients exhibit a history of sterility and peri-uterine inflammation. The fertilization of the ovum in the fallopian tube is due to a desquamated salpingitis by which the lining of the tube is deprived of its ciliary epithelium. Extra-uterine pregnancy is almost invariably tubal. The tube ruptures about the twelfth week. It may rupture through the free surface of the periphery of the tube directly into the peritoneum, as in the specimen here presented. This is a deadly accident, if the hemorrhage is not arrested by surgical means. The rupture may occur in the portion of the tube included between the folds of the broad ligament, allowing the fetal structures to escape into the cavity of the broad ligament. These latter are the cases of extra-uterine pregnancy which go on to a viable period. Extra-uterine pregnancy until very recently was not understood in its pathology, and was classified and treated as accidental hemorrhage, hematocele, etc. It is now well known that most cases of hematocele so-called are in reality cases of ectopic pregnancy. The treatment in all cases should be immediate abdominal section. The uterine appendages of both sides should be removed, inasmuch as the predisposing salpingitis is symmetrical. I have now operated in three cases within the last two years for ruptured tubal pregnancy, and all have recovered. The only safety in such a condition is immediate operation. The diagnosis before rupture is practically impossible. When rupture occurs the indications for surgical interference are as positive as in treating a wound of the brachial artery.

Dr. Geo. W. Griffiths: I can add very little to the history as already detailed. As soon as the symptoms of shock and hemorrhage appeared I advised operation. I have witnessed a great many bloody operations, and in my work as a railroad surgeon have seen many severe accidents, but I must say that when the abdomen was opened in this case and the blood gushed out it was the most formidable operation I have ever seen. I saw the patient to-day and she is entirely healed and well, though she is pale from the severe loss of blood. She went to the table and ate with the family to-day for the first time, three weeks after the operation.

Dr. I. N. Bloom: Had the symptoms been more pronounced the night you first saw her would you not have operated immediately?

Dr. McMurtry: Operation would have been immediately done had the diagnosis been absolutely positive. That is, of course, impossible before the abdomen is opened.

Dr. J. A. Ouchterlony: I do not know when I have seen a specimen and heard a report so interesting and of such great practical importance as this. It brings vividly to my mind a number of cases I have seen during the past thirty years, which were diagnosticated by myself and others with whom I was associated as pelvic hematocele, and at the same time there was always something inadequate in the diagnosis, and it seemed incomprehensible why there should be such terrific hemorrhage and such profound shock. It is a great satisfaction to know that light has been shed upon this important and perilous condition, and that we can predicate accurately the pathological condition. Cases that formerly were considered to be cases of hematocele are now known to be ruptured ectopic pregnancy. A most pleasant reflection is the fact that these cases can be so successfully managed by prompt surgical interference. It gives confidence and hope to the medical attendant, and it is a warning, and a solemn one, to lose no time in adopting the prompt course of procedure taken in the case just reported.

DR. F. LEBER: Many cases of hematocele recover by absorption, without operative interference.

DR. McMURTRY: When rupture occurs through the free surface of the tube it is a deadly accident from hemorrhage, unless treated by surgical means. If the rupture, however, takes place into the folds of the broad ligament the effusion may become absorbed, or the fetus may develop there, forming abdominal pregnancy and going on to and beyond full term. The fetal mass may break down and suppurate, discharging through the rectum or the bladder. In any contingency the safest result is secured by abdominal section. There is less danger in abdominal section according to modern methods than by taking the risk of these several terminations.

DR. T. P. SATTERWHITE: It is the first specimen of the kind I have ever seen. I agree with the essayist that it is an exceedingly difficult matter to diagnose absolutely that condition of things. In several cases, which I have seen with Dr. McMurtry, I considered his advice to open the abdomen unwise, but in every instance have been convinced that it was the correct course to pursue.

### CRUSHED FOOT.

DR. F. LEBER: I was asked to see a young man who was injured out West. It was a case of crushed foot. When he arrived at his home in Louisville he had been treated for three weeks. The foot was in a very bad condition, and I advised amputation above the ankle-joint. This was refused, and the case was treated by another physician. I was again asked to see him, and again suggested amputation, which was refused. I report this case to say that in my opinion in all such cases amputation should be done above the ankle-joint. In my opinion Chopart's amputation has never been satisfactory. I recall to mind a case left in my care by the late Dr. Cowling in which Chopart's amputation was done. It left a miserable pointed stump. I treated it for months with various devices, but never succeeded in getting a good stump. I was compelled finally to amputate. My experience during the war convinced me that none of these operations below the ankle gave such good results as amputating above the ankle.

DR. J. W. GUEST (by invitation): I had two cases of this description in the hospital. Both healed by primary union, and were discharged at the end of one month. It seems to me that in doing Chopart's amputation you save the ankle-joint as a natural joint, which is better than an artificial one. At each of these operations tenotomy was performed to prevent the stump from pointing. My experience with Chopart's amputation has confirmed that operation in my confidence. It gives a good, solid base for a foot independent of any artificial foot.

### SWEATING OF FEET.

DR. I. N. BLOOM: I wish to make a report of a case, although one case does not determine the method of treatment for a given disease. I recently had a case of sweating of the feet. The means I employed in this case were very simple. I had the patient bathe the feet in a solution of bichloride of mercury, 1 to 1,000, morning and evening. After rubbing the surface carefully so as to remove the dead epidermis macerated by the sweat, I directed the following course, which is partly though not wholly original. I had a plaster sole, partly soaked in a bichloride solution, put in the shoe, the solution being 1 to 1,000. After drying the sole and placing it in the shoe, I sprinkled it with powdered boric acid. As regards the advantage of this method of treatment there is much diversity of opinion. In this case the result was quite satisfactory. If this treatment were uniformly successful it would point to a micro-organismic origin for the disease rather than a neurological. My experience has been too short to determine, but this I know, that in many cases, especially of the lighter forms, it is of nervous origin. I have always found it much easier to cure simple hyperidrosis of the feet than the hands, and have found that Hebra's method with diachylon ointment is the only one promising any hopes of success. I have tried many other means recommended by worthy men, but always had to return to the diachylon. The inconvenience of this latter method is great, but patients bear it, or will bear any treatment that will help to get rid of the disagreeable disease. This is especially true of women.

PHARYNGEAL FISTULA.

DR. WM. CHEATHAM: I have seen recently three cases of congenital pharyngeal fistula. They all opened on the left side of the larynx. Colored fluid, such as the methyl-violet solution, injected into the fistula, passes into the pharynx; a peculiar viscid fluid, with air bubbles, escapes when pressure is made on the tract. These cases are very difficult to heal, as the course of the fistula is so sinuous, and the healing must commence at the pharyngeal end. The best method to close them is by the galvano-cautery wire.

MEETING OF JANUARY 27, 1891.

DR. J. A. OUCHTERLONY exhibited a patient, with the following remarks: The history of the case is this. The young gentleman is about eighteen years of age. A year and a half ago he began to fail in health. He came under the treatment of a gentleman who regarded the case as one of asthma. The result was that he did not improve, but continued to grow worse. He was under this doctor probably three months. Then he fell into the hands of a very careful physician, who treated him awhile and did him a great deal of good. Still the disease went on. I do not know whether it was recognized by the second physician or not. Then he was under the Blair treatment for catarrh, and from the Blair establishment he came to me in the middle of October last.

I found him with somewhat quickened breathing and pulse, temperature slightly elevated at midday, and unmistakable evidence of consolidation and incipient softening. He had lost flesh very considerably, coughed a good deal, had night sweats occasionally, and in the afternoon his hands became hot and burning. I had my friend, Mr. J. A. Flexner, examine his sputum, and found it loaded with tubercle bacilli.

While abroad this summer, I came across an article written by Dr. O. Tostensen, in Sweden, who devotes himself to the treatment of pulmonary affections. He announces that for the last two years he had been in the habit of using subcutaneous injections in cases of tuberculosis. He employed a five-gram injection, also a smaller one of one gram. When he made use of the five gram, he would make injections every other day or twice a week; but when he used one-half that size he resorted to injections every day or every other day. He always used the injection between the shoulders and on the sides, and always injected the fluid deep, not in the thickness of the skin, but would pinch up the skin and throw it right into the subcutaneous cellular tissue. A little pain is occasioned, and often some redness and swelling, but if the syringe is kept well disinfected, no abscesses follow. The formula used is the following:

| | |
|---|---|
| Acidium carbolicum niveum . . . . . | 10 |
| Menthol . . . . . . . . . . . . . . . . . | 5 |
| Vaselinum liquidum . . . . . . } | aa. 20 |
| Oleum olivarum steriliz . . . . . } | |
| Sol. bals. Peruv. 20 in petroleum benzinum, 10. | |

M. S.: One-half to a five-gram syringeful two to three times a week.

Now I began to use this solution, and although I have made some forty injections in this case I have never had a single abscess. It has been painful, but the swelling would pass away and the pain still more quickly. I have gone all around the chest. Under this treatment fever disappeared, pulse and breathing became slower, and eight pounds were gained in flesh. I regret very much to say that I have not had time to have the sputum examined to-day, but shall have it done very speedily and will report progress to the Society.

We have used the treatment at the University clinic, but without very marked results; because patients would not come regularly, and they were of a class whose hygienic surroundings were exceedingly poor.

The young gentleman recently went through an attack of acute bronchitis, undoubtedly due to taking cold, and recovered without any difficulty as readily as if it had occurred in a person entirely free from tuberculosis. The dullness has diminished in extent and degree, and pain in the chest has disappeared. No râles can be heard at present. The only internal medication he had was arsenious acid gr. 1.30 three times daily.

DR. W. H. WATHEN: At Johns Hopkins Hospital recently, where they were using the Koch lymph, I noticed that after each injection in a very short while the temperature ran up to 103° and 104°, showing marked general reaction from the use of it. They had the sphygmographic tracings that showed exactly how it affected the heart's action. This effect was so marked that it demonstrated positively that the lymph ought not to be used in any patient except under close observation.

DR. L. S. MCMURTRY: I think the report is very interesting and instructive, particularly at this time. As I understand it, it is really a constitutional treatment of tuberculosis. The injections, I presume, are introduced with a view to their being absorbed by the blood, and having an elective effect upon tubercle bacilli. And at this particular time, when the attention of the whole world is being turned to that method of treatment, it is particularly interesting. It indicates that there are many lines of treatment which most probably will lead to the same objective point, and shows, too, that we have yet to learn much before we get to the conclusion about these grades of treatment.

DR. WM. CHEATHAM: It seems to me that it is a step in the right direction. That we will have to get some substitute for the Koch treatment, on account of the unfavorable reports made of it.

DR. F. LEBER : The report was exceedingly interesting, but in my opinion was incomplete, as a correct statement ought to have been made of the condition of the sputum from week to week, as to the number of tubercle bacilli present. Sorry he did not have it done. Would like to have had the last slide compared with the original slide. The question is, was the benefit derived due to the arsenious acid or the subcutaneous injections? We have all had cases where patients have improved in the incipient state from general treatment without injections of any kind. It was supposed by the best bacteriologists that Koch's lymph was nothing but an extract of the natural tubercle itself, which it turned out to be.

DR. PETER GUNTERMANN : The report, as far as Dr. Ouchterlony has made it, is very instructive. The report as to the number of bacilli ought to have been given. I was at his office when he made several injections, and it was remarkable that there was not a sign left where former injections had been made.

DR. L. S. McMURTRY: I here present for examination by the Fellows a very large submucous uterine fibroid tumor which I removed a few days since. It is the largest tumor of this class I have ever encountered, being quite as large as a child's head at full term. The tumor had been expelled from the uterine cavity and occupied the vagina, with severe distention and pressure on adjacent organs. The pedicle was very large and was intra-uterine. The lady was thirty-eight years of age. By continuous hemorrhage she was greatly reduced and exsanguinated. In consequence of the immensity of the tumor I was compelled to remove it by sections, instead of *en masse*, in order to avoid tearing the perineum. I placed the wire of a *serreneude* around the pedicle and cut away the tumor. My friends, Drs. Griffiths and Vance, were present at the operation, and the former suggested division of the pedicle by the wire at one sitting, but I was afraid to do so. On the day after the operation, in tightening the wire, it broke, and a fearful hemorrhage resulted. The hemorrhage was very fierce and its effect was immediate upon the patient's expression. Seizing her as she lay in bed, I placed her on a table, retracted the perineum with Sim's speculum, and clamped the bleeding pedicle. Within a minute's time the bed and floor were saturated with blood. It was like *post-partum* hemorrhage. The patient is making an easy recovery.

I report the case, not only on account of the magnitude of the tumor, but particularly to warn against the dangers of hemorrhage when treated as commonly directed by authors, viz., to divide the pedicle with the wire at one sitting.

DR. F. LEBER: I have a specimen of a tumor of this kind in my possession, removed several years ago, which is larger than the one just presented. I was called to the patient by a medical friend, and found the tumor in the vagina. I believe these tumors are expelled from the uterus when comparatively small, and grow to large size in the vagina. I do not believe the danger from hemorrhage is so great as the injury to the womb. Very often a segment of the uterine tissue is included in the ecraseur and injury done in that way.

DR. J. M. MATHEWS: It has occurred to me that we meet with these polyps in the rectum. In the last two or three weeks I have removed two, but the largest I have removed was about the size of a hen's egg. The other was a soft polyp about half that size. It comes into my mind, why is it we do not have larger polyps in the rectum. The capacity is very great, and it is the same class of fibrous polyp, but they are not often met with larger than I have mentioned. Hemorrhage is the danger in polyps of the rectum, especially where the pedicle sloughs.

DR. GUNTERMANN: Last fall a year ago, when on my regular visit to the country, I met a woman who had been a patient of mine when I was practicing in the country. She had been and was under the treatment of several physicians, who had examined her, but she was bleeding all day, and every day, and had been for a year. She was near where I made my stay and requested me to see her, and on account of her having been an old patient I did so. On examination I found a little polypus about the size of a hen's egg, resting in the os. I could feel it very well. As I had no means of removing it, I requested her to come to Louisville, or I would go down and remove it. In the meantime I gave her some fluid extract of ergot, which she took in teaspoonful doses two or three times a day. The first notice I had from her she sent me the polypus, which had come away of itself.

DR. T. P. SATTERWHITE: In all operations of this kind the physicians should be prepared for hemorrhage. I assisted Dr. Geo. W. Griffiths in removing one; after it was taken away with forceps, it was as large as a child's head. There was no hemorrhage following.

DR. CHEATHAM exhibited a specimen of polypus of the esophagus, with the following remarks: An old gentleman seventy-nine years of age came to see me, saying for twelve years he had had something growing in his throat. Three years ago in voming he ejected it, caught it in his teeth, and went to Dr. Yandell, who removed it. He came to me saying it had returned; I tried with my finger to vomit him, but failed. I then tried the horsehair bougie, but failed to get the growth up. I endeavored to use the esophagoscope, but his neck was too stiff and I could not introduce it. Yesterday he came to the office with a lot of sulphate of zinc and a large twine cord, and wanted to take the zinc and vomit to eject the growth, and tie the cord around it. We gave him 25 grains of sulphate of zinc with warm water, but it failed to act promptly. I then gave him hypodermically apomorphia gr. $\frac{1}{6}$, which acted promptly, throwing the growth into the mouth where it was caught with a vulsellum. The wire of a Jarvis snare was passed over the vulsellum and growth, and pushed well down, so as to get as close as possible to the base of the polyp; it was cut off with but little difficulty. The polyp measured five inches in length, one inch in diameter, and was almost cylindrical. Close to where the wire cut through there was a considerable constriction. Mucous polyps of the esophagus are very rare.

DR. L. S. MCMURTRY: This report is exceedingly interesting. It is the first specimen of the kind I have ever seen. It illustrates incidentally what a valuable agent apomorphia is in securing prompt emesis. I would ask Dr. Cheatham his opinion as to the recurrence of this growth.

DR. WM. CHEATHAM: It is very apt to recur.

DR. W. H. WATHEN: About a month ago a lady from central Kentucky consulted me about an enlargement of the abdomen. She had a large fibroid tumor of the uterus that had probably existed for several years, but had caused no serious trouble until fifteen months before I saw her. Since then she has had excessive hemorrhage at each menstrual period, has suffered greatly from pelvic pressure, and has lost twenty-five pounds in flesh. The indications justified hysterectomy to relieve symptoms and save life. As I expected to go east in a few days to stay two weeks with my medical friends in New York, Philadelphia and Baltimore, I requested her to return to the city upon receipt of a telegram from me. On the morning of the 16th of this month she came to St. Joseph's Infirmary in a hurry to have the operation performed, and on the afternoon of the 20th an hysterectomy was done, and the uterus with a ten-pound tumor removed. The operation was complete in thirty minutes. The broad ligaments were tied off, the pedicle was secured by the neude and pins, the abdomen carefully cleansed, and the peritoneum stitched to the pedicle or neck of the uterus. She had no shock, and no untoward symptoms, until about seventy-two hours, when her pulse became rapid and she could not retain anything in her stomach. Her pulse finally reached 150 per minute, but hei temperature was never above 100°. In a hundred and ten hours she died, apparently from septic infection; but as she had comparatively no fever, death may have been caused by intestinal obstruction from paresis of the bowels. Cases of this sort have been reported where no cultures could be made from the peritoneum or its secretions. She could not retain salines, and I gave her one grain of calomel every hour, but neither gas nor fecal matter would pass. If death was caused by septic infection, the point I wish to especially emphasize is the difficulty in excluding septic matter in operations involving the peritoneum. This is the only case of septic infection I have had in my laparotomies in eighteen months, and the surrounding conditions were never apparently more favorable, and I have never been more careful. The instruments and sponges were carefully cleansed in boiled water, and neither my nurse nor myself had been exposed to septic influences. The untimely death of this woman has so impressed me with the necessity of absolute surgical cleanliness in laparotomy work, and of my moral and professional responsibility, that I have had prepared a room in an addition to one of our infirmaries for such operations, with all modern aseptic appliances and conveniences, such as plate-glass top-tables, porcelain-lined pans, sterilizer, etc.

